# Feasibility of mail-based biospecimen collection in an online preconception cohort study

**DOI:** 10.3389/frph.2022.1052231

**Published:** 2023-01-09

**Authors:** Martha R. Koenig, Amelia K. Wesselink, Andrea S. Kuriyama, Alina Chaiyasarikul, Elizabeth E. Hatch, Lauren A. Wise

**Affiliations:** Boston University School of Public Health, Department of Epidemiology, Boston, MA, United States

**Keywords:** biospecimen collection, internet cohort, preconception, comparison, methods

## Abstract

**Background:**

Prospective cohort studies that enroll participants before conception are crucial for deepening scientific understanding of how the preconception environment influences reproductive outcomes. While web-based research methods provide efficient and effective strategies to collect questionnaire-based data, few of these studies incorporate biospecimen collection, which can enhance the validity of exposure assessment. There is limited literature on the feasibility and cost-effectiveness of collecting biospecimens in web-based preconception cohort studies.

**Methods:**

We evaluated the feasibility and cost-effectiveness of in-clinic and mail-based biospecimen collection in Pregnancy Study Online (PRESTO), a North American web-based preconception cohort study. Both members of the couple were eligible to participate if their conception attempt time was ≤3 months at enrollment. We invited study participants from the Boston, MA and Detroit, MI metropolitan areas to attend a study visit and provide urine and blood (hereafter “in-clinic protocol”). We invited all other participants to complete mail-based collection of urine and blood spots (hereafter “mail-based protocol”). We compared overall consent and protocol completion rates, demographic characteristics of those who consented and completed either of the protocols, and costs between mail-based and in-clinic protocols for biospecimen collection. Finally, we described logistical challenges pertaining to reliance on mail-based delivery of time-sensitive biospecimens compared with in-clinic methods.

**Results:**

During January 2022-July 2022, 69% of female participants (134/195) and 42% of male participants (31/74) consented to participate in the mail-based protocol. Consent rates for the in-clinic protocol were 39% for female participants (289/739 during March 2014-July 2022) and 25% for male participants (40/157 during March 2017-July 2022). Participants who consented to participate were generally of higher socioeconomic position than non-participants. Deviations from the protocol occurred more frequently within the mail-based protocol but were easily corrected. The cost per participant enrolled was similar across protocols (mail-based: $276.14 vs. in-clinic: $270.38).

**Conclusions:**

Our results indicate that mail-based collection of biospecimens may create opportunities to recruit a larger and more geographically diverse participant population at a comparable cost-per-participant enrolled to in-clinic methods.

## Introduction

A growing number of prospective cohort studies implement web-based methods for recruitment, follow-up, and data collection. Web-based cohort studies have the potential to recruit more geographically diverse study populations at a lower cost per participant enrolled when compared with traditional cohort studies (1–3). The use of web-based questionnaires has been associated with a greater likelihood of participation in survey research overall, and questionnaire completion among enrolled participants (4, 5) Furthermore, they reduce participant burden by eliminating travel to clinics and minimizing interactions with study staff (3, 6). However, web-based methods require technical expertise to develop, maintain, and secure complex recruitment methods, questionnaires, and databases (7, 8). Researchers may struggle to develop rapport among participants without face-to-face interaction, possibly resulting in lower participant engagement (9).

Biospecimen collection in epidemiologic research can enhance the validity of exposure assessment by providing more direct biomarkers of environmental toxicants, hormones, nutrients, genomic characteristics, and general health status (e.g., lipid profile, iron status) relative to self-reported data (10–12). Traditionally, biospecimen collection takes place within a clinic, a method that maximizes sample quality control, but may be burdensome for the participant to schedule and travel to, thereby impacting recruitment (13). Modern technologies have increased potential for rapid mailing and at-home sample collection, a method that may reduce participant burden and improve recruitment, but may introduce new challenges related to sample collection, sample quality, and logistics pertaining to shipment (14) Despite the many advantages of biospecimen collection for exposure assessment, there is a lack of literature about how web-based studies can feasibly incorporate biospecimen collection, and what methods of biospecimen collection are most acceptable for participants and feasible and productive for researchers.

Prospective cohort studies that enroll participants before conception are crucial for deepening scientific understanding of how the preconception environment influences reproductive outcomes (15). In this report, we describe the feasibility and cost-effectiveness of biospecimen collection in Environmental Pregnancy Study Online (E-PRESTO), a substudy nested within Pregnancy Study Online (PRESTO), a North American web-based preconception cohort study. Specifically, we compare mail-based biospecimen collection and traditional in-clinic biospecimen collection overall consent and completion percentages, and characteristics of those who consented to and completed the in-clinic and mail-based protocols. We also provide a cost comparison of both protocols. Finally, we describe logistical challenges pertaining to reliance on mail-based delivery of time-sensitive biological specimens.

## Materials and methods

### Study design

PRESTO is an ongoing web-based prospective cohort of couples residing in the United States and Canada that began in June 2013. Methods of recruitment, enrollment, and primary data collection are described in detail elsewhere (3). Briefly, we recruit participants during the preconception period primarily using advertisements on social media such as Facebook and Instagram. Advertisements are designed to reach all geographic regions in the United States and Canada. Eligible participants self-identify as female, are aged 21–45 years, and are actively trying to conceive without the use of fertility treatments. Immediately after enrollment, female participants are encouraged to invite their male partners aged ≥21 years to participate. Both partners complete a baseline questionnaire where they provide comprehensive data regarding socio-demographics, behavioral and lifestyle factors, diet, and medical and reproductive history. Female participants are invited to complete bimonthly follow-up questionnaires for 12 months or until pregnancy is reported, whichever occurs first.

### E-PRESTO in-clinic biospecimen collection

In March 2014, we began recruiting and enrolling PRESTO participants into Environmental Pregnancy Study Online (E-PRESTO), a sub-study designed to assess the association of environmental chemical exposures with reproductive health. Eligible participants are both members of the couple who completed the PRESTO baseline questionnaire, had been trying to conceive for ≤3 months at enrollment, and lived or worked in the Boston, MA or (beginning in 2017) the Detroit, MI metropolitan areas. Eligible participants were invited *via* an automated email system within one hour after completion of the baseline questionnaire.

After providing online consent to the in-clinic protocol, we invited participants to schedule a clinic visit to provide baseline biospecimen samples. Prior to the appointment, participants received reminder emails and were given instructions as to the clinic location and the site for parking. During the clinic visit, participants collected a urine sample, and a trained phlebotomist collected intravenous blood samples. At the end of the in-clinic visit, participants received a $50 gift card. In October 2019, we began sending participants home with a urine collection kit (a large biohazard bag containing a box with instructions, a log form, and three urine collection cups, each within their own smaller biohazard bags). Participants were asked by study staff to collect urine samples over a 12-day period (on days 3, 6, and 9-post clinic visit) and store all collected urine samples in their home freezer. Participants received email reminders on days 3, 6, and 9. They were prompted to select a date, time, and location for the samples to be picked up by either study staff or through the utilization of a ride-share app. Once the three additional urine samples were retrieved, the participant was compensated an additional $20 through an online link to a gift card. The biospecimens were then brought to the Boston University laboratory for processing and storage at −80^o^ Celsius (i.e., pooling of urine across days 0, 3, 6, and 9).

### E-PRESTO mail-based biospecimen collection

Beginning in January 2022, we extended the E-PRESTO protocol to include mail-based biospecimen collection for all eligible participants who lived within the contiguous United States but outside the Boston or Detroit metropolitan areas. We invited eligible participants *via* email, within one hour after enrollment into the main study. The goals of mail-based biospecimen collection were to expand the geographic diversity of study participants and reduce participant burden. Participation in the protocol involved receiving a biospecimen collection kit through the mail, collecting four urine specimens (three days apart over the course of a 12-day period), freezing urine samples in their home freezer, as well as providing blood spots, obtained by pricking one's finger with a lancet and sampling the blood onto a collection card.

Study staff mailed a kit containing all study materials to consenting participants using two-day shipping. The kit contained the following materials: insulation, packing tape, four small ice packs, instructions for completing the protocol and mailing back the biospecimens, an overnight return shipping label, a blood spot collection kit (containing instructions and examples of “good quality” blood spots, two small lancets, blood spot collection card, and a small plastic bag with desiccant), and a urine collection kit (a large biohazard bag containing a box wrapped with absorbent pads with instructions, a log form, and four urine collection cups, each within their own smaller biohazard bag). After receiving their kit in the mail, participants were instructed to click a link in their email, which triggered morning reminders *via* email to collect urine samples on days 3, 6 and 9 and to collect blood spots if they had not done so already. We encouraged participants to email study staff with any questions. On day 10, we sent participants an automated email to schedule a drop off at a mail-carrier or a mail-carrier at-home pick-up for the return of their biospecimen collection package. For both options, participants could only select Monday-Thursday so that a staff member could receive it on a regular business day. According to participant selections, staff scheduled a mail-carrier pick-up and prepared for the arrival of the biospecimens.

When study staff received the mail-based collection kit at Boston University, they ensured the log form was included and completed. Participants received $40 compensation for completing the blood spot collection and $20 compensation for completing the urine collection *via* gift card links sent *via* email. The biospecimens were then brought to the Boston University laboratory for processing and freezer storage at −80° Celsius.

For both the in-clinic and mail-based protocols, female participants who conceived during the study period were invited to complete the protocol again during their second trimester of pregnancy (*via* automated email invitation). Male partners were invited to participate only once during preconception.

**Figure 1:**
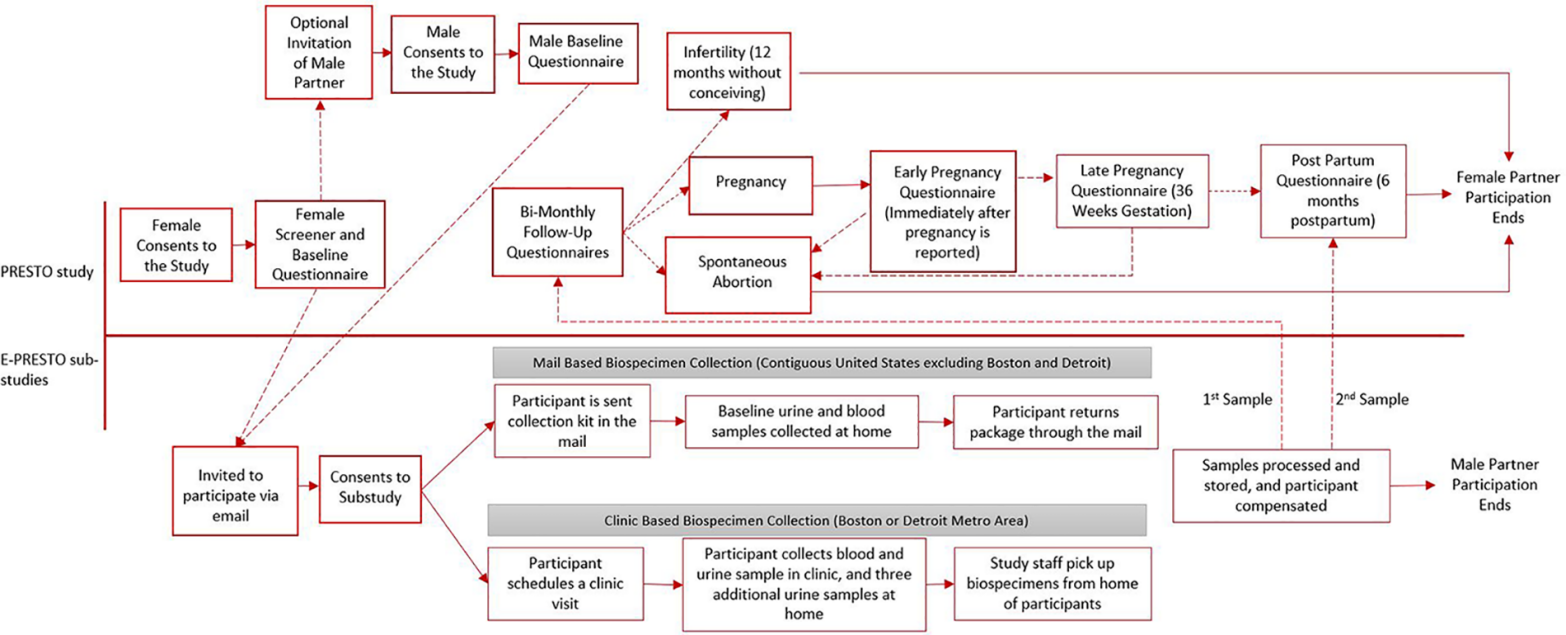
Timeline of study procedures and data collection for PRESTO and E-PRESTO sub-studies.

Both in-clinic and mail-based E-PRESTO protocols were approved by the institutional review board at Boston University Medical Campus. Participants completed separate online consent forms to participate in PRESTO and E-PRESTO studies. Figure 1 shows a timeline of procedures and data collection methods for both studies.

### Data analysis

We provided descriptive statistics on consent and completion rates for the in-clinic and mail-based E-PRESTO sub-studies. We compared sociodemographic, lifestyle, medical and reproductive characteristics across those who did not consent, those who consented but did not complete the protocol, and those who completed the protocol, stratified by sex of the participant. We also examined consent and completion rates by nine geographic regions for the mail-based study. Recruitment for both protocols remains ongoing, so the sample sizes and percentages listed in all tables or figures relating to consent and completion rates within sub-studies were restricted to those who consented 30 days before the analysis to ensure they could complete the full protocol. In this analysis, we do not report on pregnancy-related visits. All analyses were descriptive in nature and were performed using SAS version 9.4 (SAS Institute, Cary, NC).

To estimate the cost of collecting biospecimens *via* mail vs. clinic, we restricted our comparison to the urine collection component because it was identical across both methods (i.e., 4 urine cups collected during a 12-day period).

Study staff reviewed email communications and notes from all participants in both the mail-based and in-clinic who completed the protocols to document the number and type of protocol deviations within each protocol. Each category of deviation was given a frequency, assigned to a step in the protocol, an actor responsible (when applicable), as well as the standard solution to correct the deviation.

## Results

**Figure 2:**
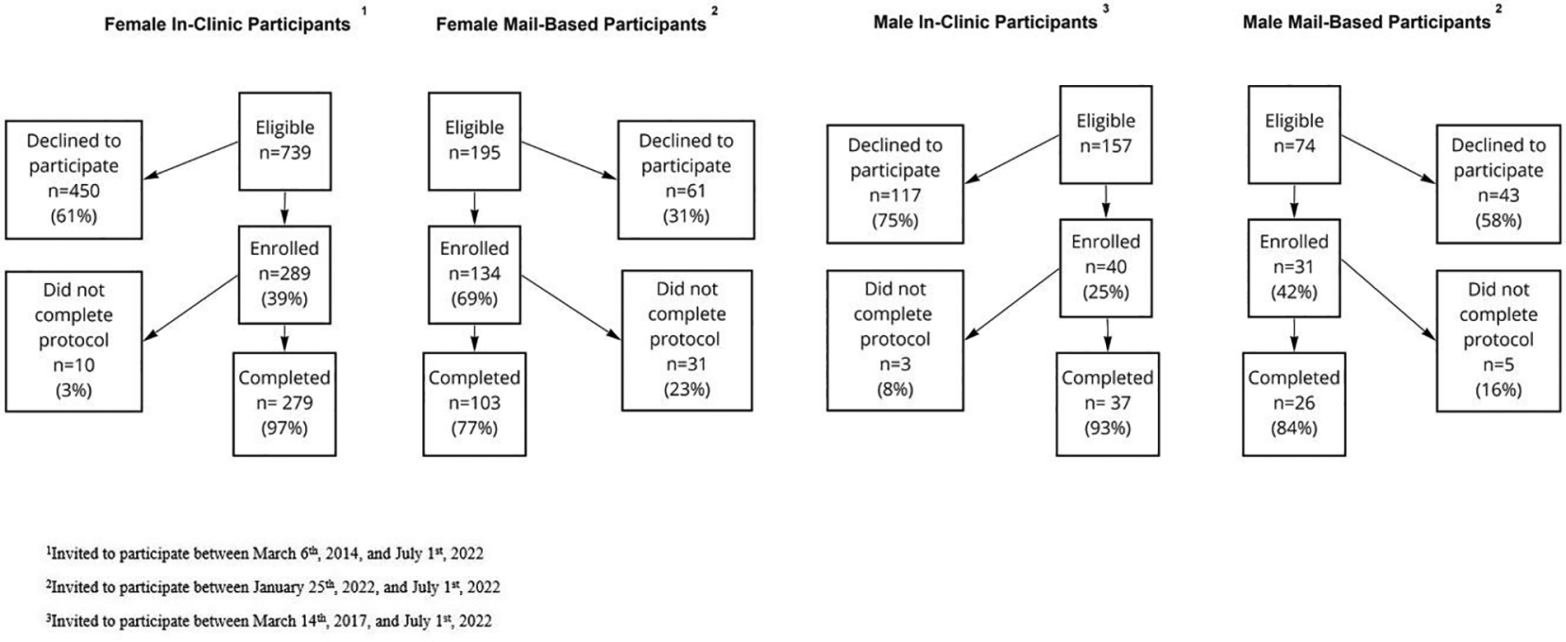
Flowchart of exclusions for participants in the mail-based and in-clinic E-PRESTO sub-studies.

During March 2014 through July 2022, 39% (289/739) of eligible female participants and 25% (40/157) of eligible male participants from the Boston, Massachusetts and Detroit, Michigan metropolitan areas consented to participate in the in-clinic protocol. During January 2022 through July 2022, 69% (134/195) of eligible female participants and 42% (31/74) of eligible male participants consented to participate in the mail-based protocol. Among those who consented to the study, Male and female participants had similar completion rates within the mail-based protocol (84% vs. 77% respectively) and the in-clinic protocol (93% vs. 97%, respectively) (Figure 2).

**Figure 3:**
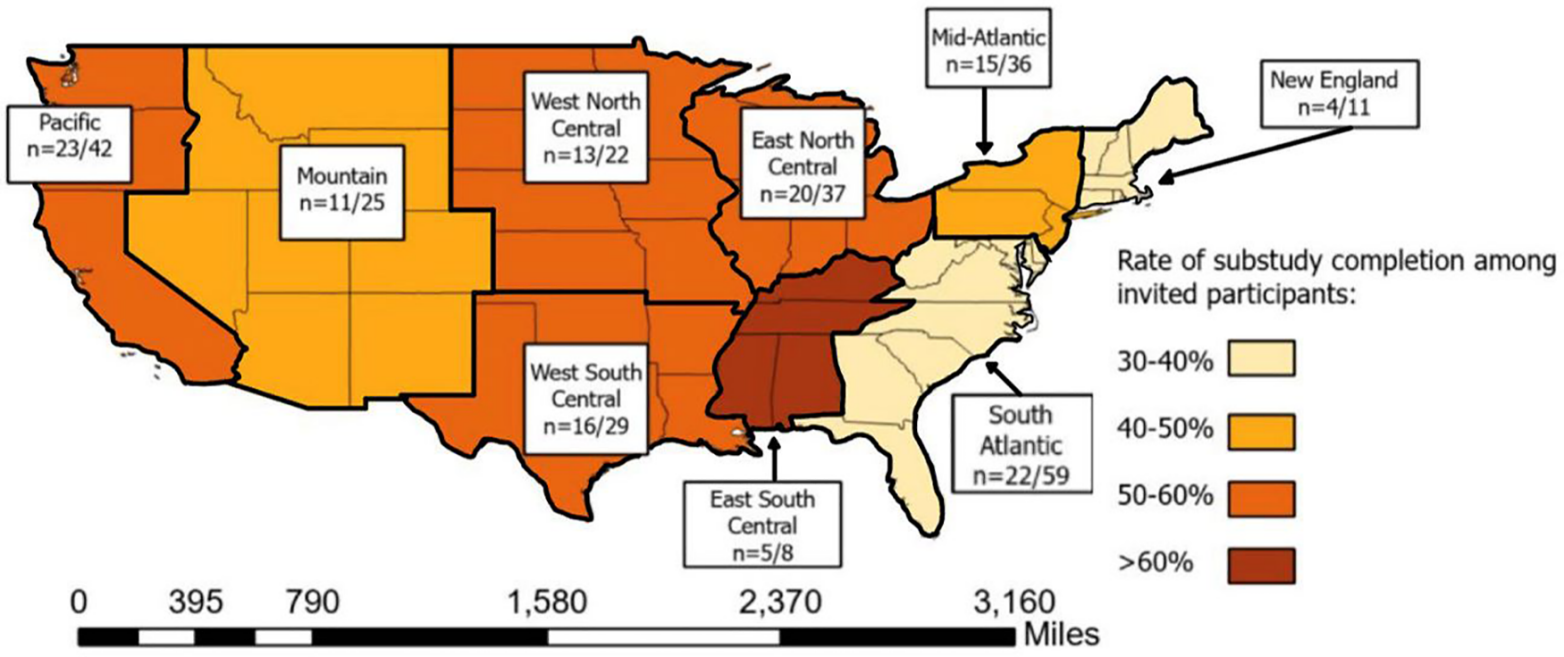
Density of participants completing the mail-based protocol across 9 geographic subregions of the United States of America.

The mail-based protocol recruited participants from all 9 regions recognized by the United States. Census (Pacific, Mountain, West North Central, West South Central, East North Central, East South Central, South Atlantic, Middle Atlantic and New England). Most participants who completed the protocol resided in the Pacific (*n* = 23), South Atlantic (*n* = 22) and East North Central (*n* = 20) regions. The regions of New England and East South Central were home to the fewest participants, with ≤5 completing the protocol in each region. Completion percentages ranged from 63% in the East South-Central Region to 36% in New England and 37% in the South Atlantic (Figure 3).

The median ages of female and male participants completing the in-clinic protocol were 32 and 33 years, respectively. Within the mail-based protocol, the median ages of female and male participants who completed the protocol were 31 and 32 years, respectively. Female participants reporting a history of infertility were more likely to complete the mail-based study (11%) than those who did not consent to the protocol (3%). Males who had a previous conception were less likely to participate in both the in-clinic and mail-based protocols (40% vs. 28% for the in-clinic protocol and 52% vs. 28% for the mail-based protocol). Characteristics of participants who consented but did not end up completing the protocol were similar to characteristics of participants who consented and completed the protocol: both sets of participants were more likely to have higher educational attainment and income and identify as non-Hispanic white compared to PRESTO participants who did not consent to an E-PRESTO protocol (Table 1).

**Table 1:** Characteristics of enrolled E-PRESTO participants stratified by sex, protocol, consent, and successful completion of the protocol.

Characteristic	Female participants						Male participants					
	In-clinic protocol			Mail-based protocol			In-clinic protocol			Mail-based protocol		
	Did not consent^a^ (*n* = 450)	Consented but did not complete^b^ (*n* = 10)	Consented and completed^c^ (*n* = 279)	Did not consent (*n* = 61)	Consented but did not complete (*n* = 31)	Consented and completed (*n* = 103)	Did not consent (*n* = 117)	Consented but did not complete (*n* = 4)	Consented and completed (*n* = 37)	Did not consent (*n* = 43)	Consented, but did not complete (*n* = 5)	Consented and completed (*n* = 26)
Age (years) (Median, IQR^d^)	31 (28–33)	31 (29–32)	32 (29–34)	29 (28–34)	32 (28–35)	31 (28–34)	32 (30–35)	31 (28.5–34.5)	33 (31–37)	32 (30–38)	31 (30–33)	32 (31–35)
Attempt Time at Study Entry (cycles) (Median, IQR)	1 (1–1)	1 (1–1)	1 (1–2)	1 (1–2)	1 (1–2)	1 (1–2)	1 (1–2)	1.5 (1–2)	1 (1–1)	1 (1–2)	2 (1–2)	1 (0–1)
Education (years), %												
≤12	1.8	5.6	0.7	0.0	12.9	1.0	6.8	25.0	5.1	4.7	0.0	0.0
13–15	11.6	11.1	6.1	16.4	16.1	7.8	13.7	25.0	5.1	27.9	60.0	11.5
16	24.9	27.8	27.8	29.5	16.1	29.1	37.6	0.0	25.6	25.6	0.0	42.3
≥17	61.8	55.6	65.5	54.1	54.8	62.1	41.9	50.0	64.1	41.9	40.0	46.2
Household income (USD^e^/year), %												
<$50,000	4.8	5.6	4.0	6.6	19.4	7.8	2.6	25.0	2.6	9.5	20.0	0.0
$50,000–$99,999	21.7	29.4	24.4	29.5	29.0	24.5	21.1	25.0	20.5	26.2	40.0	11.5
$100,000–$149,999	35.7	29.4	33.7	21.3	19.4	31.3	36.0	0.0	20.5	30.1	40.0	23.1
≥$150,000	37.8	35.3	38.0	42.6	32.3	36.3	40.4	50.0	56.4	33.3	0.0	53.8
Race/ethnicity, %												
Non-Hispanic white	83.8	72.2	80.4	85.3	77.4	87.4	81.2	100.0	79.5	72.1	60.0	80.8
Non-Hispanic Black	2.4	5.6	5.0	3.3	6.5	0.97	5.1	0.0	5.1	0.0	0.0	0.0
Non-Hispanic Asian	4.7	5.6	3.9	1.6	0.0	3.9	2.6	0.0	5.1	0.0	20.0	3.9
Non-Hispanic other^f^	2.4	0.0	3.2	0.0	0.0	1.9	2.6	0.0	7.7	4.7	20.0	7.7
Hispanic	6.7	16.7	7.5	9.8	16.1	5.8	8.6	0.0	2.6	9.3	0.0	7.7
Urbanicity^g^, (%)												
Rural	0.7	0.0	0.0	3.3	3.2	2.9	0.0	0.0	0.0	4.7	0.0	3.9
Urban Cluster	0.7	0.0	0.0	13.1	9.7	8.7	0.9	0.0	0.0	16.3	0.0	11.5
Urban	98.7	100.0	100.0	83.6	87.1	88.4	99.2	100.0	100.0	79.1	100.0	84.6
Employed, (%)	92.9	83.3	93.2	91.8	87.1	89.3	94.0	75.0	94.9	95.4	100.0	96.2
Hours of Work/week (Median, IQR)	40 (35–40)	40 (20–40)	40 (36–41)	40 (36–40)	40 (35–40)	40 (32–40)	40 (40–50)	40 (20–42.5)	40 (40–45)	40 (40–45)	46 (42–50)	41 (40–48)
Current Smoker, (%)	2.5	0.0	1.1	1.6	0.0	1.0	1.7	0.0	0.0	4.7	0.0	0.0
Parous, (%)	43.7	61.1	42.3	47.5	41.9	45.6	—	—	—	—	—	—
Ever Impregnated a Partner (%)	—	—	—	—	—	—	40.0	25.0	28.2	52.4	0.0	28.0
History of Infertility (%)	3.8	5.6	3.6	3.3	0.0	10.7	—	—	—	—	–	—

^a^ Participant was eligible to participate in the protocol but did not consent to participate after invitation *via* email.

^b^ Participant consented to participate but did not complete the protocol by either not showing up to the in-clinic appointment, or coordinating the return of biospecimens.

^c^ Participant collected all biospecimens and was compensated for their efforts.

^d^ Interquartile Range.

^e^ United States Dollars.

^f^ Other includes those who identify as mixed-race, Native American or Pacific Islander, and Middle Eastern or North African.

^g^ “Urban” refers to residing within a United States census tract with 50,000 people or more, “Urban Clusters” refers to residing within a United States census tract of at least 2,500 and less than 5,000 people “Rural” encompasses all census tracts not included within an urban area.

Across six different stages of the in-clinic protocol, we identified 11 categories of deviations and three different actors in those deviations including participants, laboratory staff, study staff and occasions where the deviations were unattributable. Among all completed in-clinic visits, 30% involved at least one protocol deviation. The most common protocol deviations were insufficient sample collection at the clinic visit (*n* = 34) and a request to reschedule the time which urine samples collected at the home were picked up (*n* = 16).

**Figure 4:**
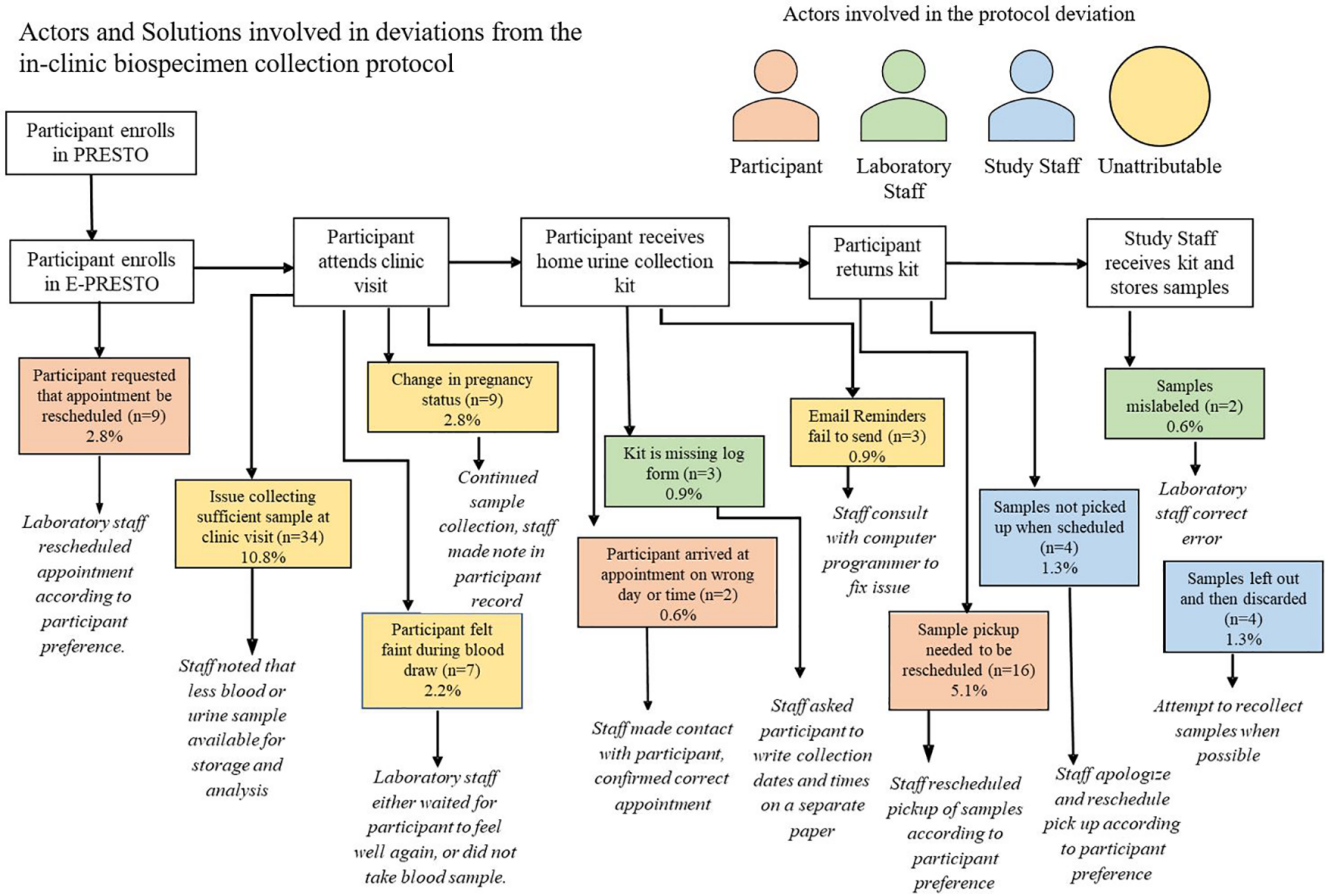
Actors and solutions involved in deviations from the in-clinic biospecimen collection protocol.

**Figure 5:**
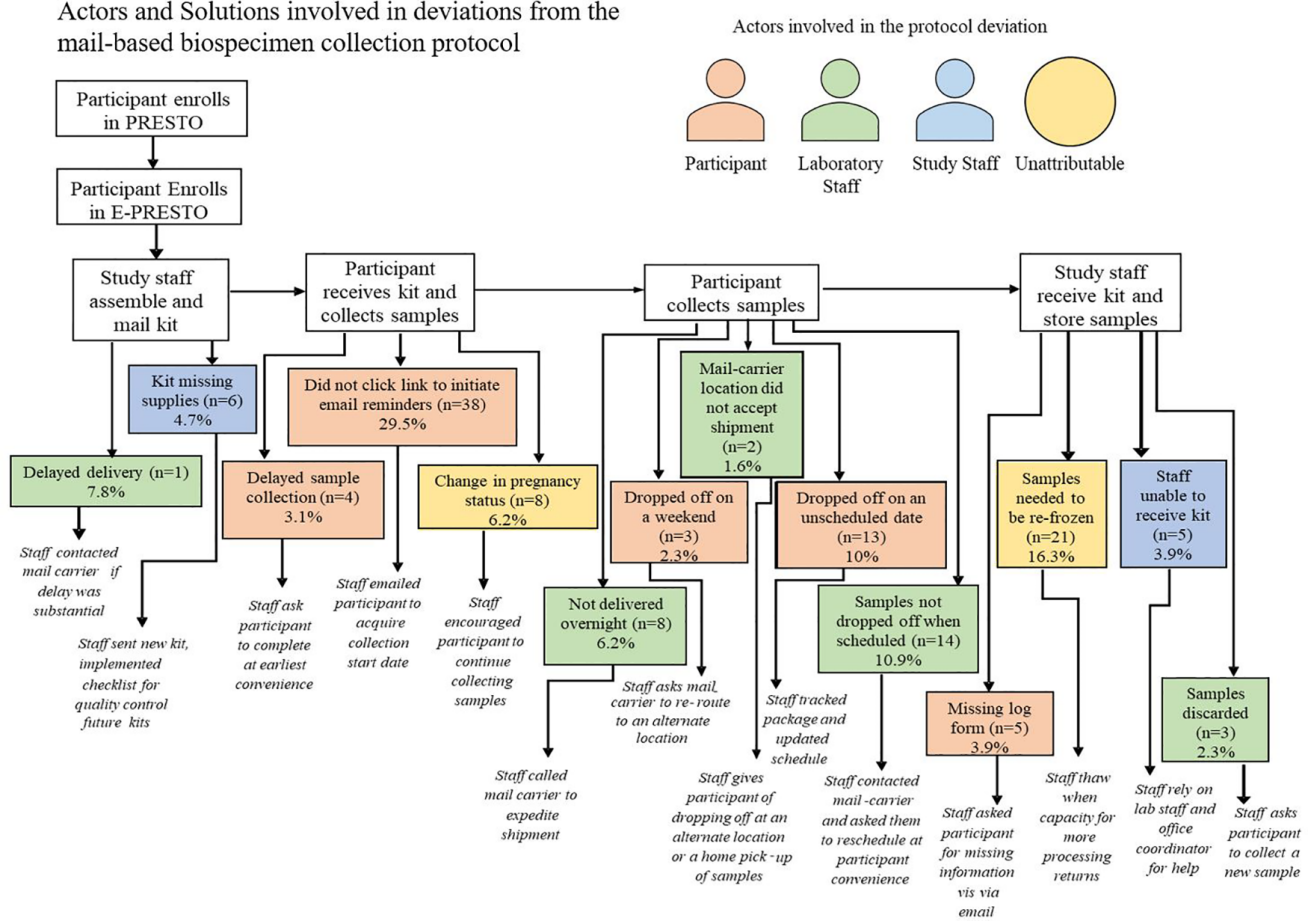
Actors and solutions involved in deviations from the mail-based biospecimen collection protocol among participants who complete the protocol.

Across six different stages of the mail-based protocol, we identified 14 categories of deviations and three different actors in those deviations including the mail-carrier, participant, study staff and occasions where the deviation was unattributable. Among all completed biospecimen collection kit returns within the mail-based protocol 47% involved at least one protocol deviation. The most common protocol deviations were participants not initiating email reminders to collect biospecimens and return their package although their package was successfully returned (*n* = 38), needing to re-freeze samples which arrived thawed due to reaching the laboratory processing capacity for the week (*n* = 21), and the mail-carrier not picking the package up when scheduled for the return delivery back to study staff (*n* = 14). Insufficient blood-spot collection from participants in the mail-based protocol was also a frequent occurrence (8%) but is beyond the scope of this paper. Each deviation for both the in-clinic and mail-based protocols had a standard response from study staff that was intended to re-align the protocol back to its intended sequence (Figures 4,5).

Estimated costs for the in-clinic and mail-based urine collection protocols were nearly identical (mail-based: $276.14 vs. in-clinic: $270.38). The price of two-day shipping costs (for delivery of the biospecimen collection kit to the participant) ranged from $8.39 to $51.68 (average: $22.94), and the cost of overnight shipping (for return of biospecimens from the participant to the study staff) ranged from $8.13 to $53.25 (average: $26.77), depending on geographic region of residence. We estimated average shipping costs to be $49.71 per participant. The average cost of supply for the mail-based kits was $7.83 per kit. We estimated study staff time to be one hours' worth at $22.60/hour for the mail-based study, which includes package assembly, shipment, record keeping, coordination of the return, sample receipt and payment, and transferring of samples for drop-off and processing. Within the in-clinic protocol, effort required by study staff was reduced and estimated to be 30 min' worth of work at $22.60/hour ($11.30 total). This effort includes coordination of return of the biospecimen kit, record keeping, participant compensation, and transferring samples for drop off at the laboratory. The in-clinic protocol included higher laboratory costs due to more involvement of clinic staff in scheduling and collecting of samples ($54 vs. $22). For the in-clinic protocol, participants were given a $7 voucher for on-site parking during their visit, and at the completion of their home urine collection period, a rideshare app retrieved their samples with a cost ranging between $8.53 and $46.79 and averaging $26.08 per participant (Table 2).

**Table 2:** Cost analysis for in-clinic and mail-based urine collection.

Item	In-clinic protocol (Boston)	Mail-based protocol
Recruitment	$125	$125
Participant compensation	$20	$20
Collection and processing supplies^a^	$25	$25
Postage^b^	n/a	
Two-day delivery		$22.94 ($8.39–$51.68)
Overnight return		$26.77 ($8.13–$51.68)
Supplies for mailing^c^	n/a	$7.83
Study staff compensation	($26.60 × 0.5 h)	($26.60 × 1.0 h)
Laboratory Costs		
Scheduling	$17	n/a
Baseline Urine Collection	$15	n/a
Processing of Urine	$22	$22
On-site parking (2 h)	$7	n/a
Ride-app cost for urine pick-up^d^	$26.08 ($8.53–$46.79)	n/a
Total	$270.38	$276.14
Cost Difference	$5.76	

^a^ Four 4-ounce urine collection cups in biohazard plastic bags, inside a cardboard box in a large biohazard plastic bag.

^b^ Costs are higher when further from Boston and in more remote locations.

^c^ Includes a cardboard box, insulation materials, ice-packs, and packaging tape.

^d^ Cost is dependent on distance from Boston University School of Public Health, and surge pricing (ride-share costs are more expensive during high-demand times of the day).

### Discussion

In a web-based preconception cohort study, both mail-based and clinic-based biospecimen collection approaches were acceptable to participants and feasible for participants, study staff, and investigators. Among invited participants, those who consented to and completed either of the protocols had higher socioeconomic position than those who did not consent. A cost-comparison revealed that the costs of in-clinic and mail-based urine collection were negligibly different. Approximately 30% of those completing the in-clinic protocol and 47% those completing the mail-based protocol encountered at least one deviation from the protocol; however, these deviations were easily addressed.

In the present study, the clinic protocol was entirely urban by design, while the mail-based protocol recruited participants residing in areas of varying levels of urbanicity across the nation. This may partially explain varying participant characteristics across the two protocols. PRESTO participants have higher educational attainment and annual income than the general population, which may limit the generalizability of our findings. Other studies have not identified demographic characteristics that may influence participation using mail-based biospecimen collection methods (16, 17).

Of interest, the Florida Health and Ancestry Survey recruited a more representative sample of their population of interest using respondent panels or random digit dialing but reported lower consent and completion rates of mail-based biospecimen collection compared with convenience samples at cancer centers (18). The investigators are aware of one other prospective web-based study that included mail-based biospecimen collection among adults with irritable bowel disease (19). Consent and completion rates were 72% and 40%, comparable to the consent rates in the present study for females and males, respectively.

Overall, male PRESTO participants were less likely to provide consent to participate in biospecimen collection (in-clinic and mail-based) than female participants. This may be due a belief that their contributions to fertility research are less important than those of their female partners, or insecurities related to participation in fertility research (20). Males who completed either biospecimen collection protocol had higher educational attainment and income than those who did not consent or complete either protocol and were less likely to have ever impregnated a partner than those who did not consent. This observation contrasts with published findings from the semen testing protocol within PRESTO where male history of having impregnated a partner was comparable between those who did and did not consent (21). Within the E-PRESTO population, male participants who had already impregnated their partners could have been more confident in their fertility and less motivated to participate.

It is unclear which variety of factors motivate individuals to contribute biospecimens for research. Time associated with sample collection has been noted by other researchers to be a burden to successful collection of samples (22). Rates of consent within our mail-based protocol suggest that the reduced participant burden of collecting samples within one's home without needing to visit a clinic is a key motivator for providing biospecimen samples. The in-clinic protocol created a greater degree of participant burden resulting from time (driving to and from the clinic, and attending the appointment), schedule disruption (to work, childcare, or other obligations), and effort (scheduling and driving). While other investigators have reported that higher monetary compensation is associated with greater participation, we could not compare the influence of compensation being a motivating factor because compensation levels were similar across both protocols, apart from travel expenses for the in-clinic protocol (e.g., fuel, mileage, and tolls) 23).

Overall, study costs for urine collection were similar across mail-based and in-clinic protocols albeit costs for the mail-based protocol were a few dollars more. The mail-based protocol was more flexible and resulted in a greater overall rate of recruitment during a shorter period from a more geographically-diverse population. Higher rates of non-completion within the mail-based study come with a cost not considered in our comparison. Costs for two-day delivery, shipping materials, collection materials and person-time by study staff were incurred when participants did not return samples to us after consenting to the study, equating to a loss of approximately $69.07 per kit (not shown). This limitation may be balanced by strength of this method to maximize the biospecimen collection data given a fixed number of enrolled participants in PRESTO. Therefore, its adoption may be highly attractive and valuable in epidemiologic research when loss to follow-up can be minimized, as the cost of implementation is not meaningfully different from collecting samples within a clinic.

Deviations within the mail-based protocol occurred frequently and were often attributable to the need to rely on a third-party mail-carrier and being in the pilot testing stages of the protocol. Study staff were able to quickly recognize and respond to protocol deviations in both protocols to collect quality biospecimens in an efficient manner, which rarely led to the need for biospecimens to be discarded. Nevertheless, mail-based delivery prolongs the amount of time during which the sample is not temperature-controlled and increases the need to re-freeze samples upon receipt. Such protocol deviations may impair the quality of the sample when it is eventually analyzed (24). Protocol deviations took place less commonly within the in-clinic protocol, which reflects the advantages of traditional biospecimen collection where investigators rely on contact between staff and participants for in-person sample collection within a select geographic area. Fewer protocol deviations, especially as they relate to sample transport and storage, not only reduce burden for study participants and staff of needing to correct deviations but may also improve the quality of the sample when it is eventually sent for analysis. We did not assess sample quality in our study, so the degree to which these samples were compromised by uncontrolled temperatures is unknown.

Detailed information regarding participant characteristics and associated costs of implementation are strengths of our study. Study limitations include small numbers (precluding any meaningful statistical analyses), limited study period in the mail-based study (6 months) compared with the in-clinic study (8 years), and lack of detailed data related to participants motivations for participating in biospecimen collection research. Future research should query participants about the motivators and barriers to participating in various methods of biospecimen collection. In summary, participants in a national web-based prospective preconception cohort study who were invited to provide biospecimen samples through the mail had relatively high rates of consent and protocol completion, with costs of implementation comparable to in-clinic biospecimen collection among a cohort of couples attempting to conceive. Mail-based biospecimen collection may allow investigators involved in longitudinal research studies to engage in sustainable and continuous sample collection from participants who do not reside near a clinic, cannot make the time to attend an appointment, or prefer to collect samples within the comfort of their own home.

## Data Availability

The raw data supporting the conclusions of this article will be made available by the authors, without undue reservation.
