# Corrigendum: Feasibility of mail-based biospecimen collection in an online preconception cohort study

**DOI:** 10.3389/frph.2025.1582697

**Published:** 2025-03-13

**Authors:** Martha R. Koenig, Amelia K. Wesselink, Andrea S. Kuriyama, Alina Chaiyasarikul, Elizabeth E. Hatch, Lauren A. Wise

**Affiliations:** Boston University School of Public Health, Department of Epidemiology, Boston, MA, United States

**Keywords:** biospecimen collection, internet cohort, preconception, comparison, methods

A Corrigendum on Feasibility of mail-based biospecimen collection in an online preconception cohort study By Koenig MR, Wesselink AK, Kuriyama AS, Chaiyasarikul A, Hatch EE and Wise LA (2023). Front. Reprod. Health. 4. doi: 10.3389/frph.2022.1052231

**Error in Figure/Table**

In the published article, there was an error in Table 1 as published. During the proofing stage, we did not notice that the typesetter had conflating the categories of “mail-based protocol” and “male participants”. The corrected version ensures that “Mail-based protocol” is properly aligned under both female and male participants, clearly distinguishing participant groups and protocol types. The corrected Table 1 and its caption appear below.

Published Version:

**Table 1:** Characteristics of enrolled E-PRESTO participants stratified by sex, protocol, consent, and successful completion of the protocol.

Characteristic	Female participants						Mail-based protocol					
	In-clinic protocol			Male participants			In-clinic protocol			Mail-based protocol		
	Did not consent^a^ (*n* = 450)	Consented but did not complete^b^ (*n* = 10)	Consented and completed^c^ (*n* = 279)	Did not consent (*n* = 61)	Consented but did not complete (*n* = 31)	Consented and completed (*n* = 103)	Did not consent (*n* = 117)	Consented but did not complete (*n* = 4)	Consented and completed (*n* = 37)	Did not consent (*n* = 43)	Consented, but did not complete (*n* = 5)	Consented and completed (*n* = 26)
Age (years) (Median, IQR^d^)	31 (28–33)	31 (29–32)	32 (29–34)	29 (28–34)	32 (28–35)	31 (28–34)	32 (30–35)	31 (28.5–34.5)	33 (31–37)	32 (30–38)	31 (30–33)	32 (31–35)
Attempt Time at Study Entry (cycles) (Median, IQR)	1 (1–1)	1 (1–1)	1 (1–2)	1 (1–2)	1 (1–2)	1 (1–2)	1 (1–2)	1.5 (1–2)	1 (1–1)	1 (1–2)	2 (1–2)	1 (0–1)
Education (years), %												
≤12	1.8	5.6	0.7	0.0	12.9	1.0	6.8	25.0	5.1	4.7	0.0	0.0
13–15	11.6	11.1	6.1	16.4	16.1	7.8	13.7	25.0	5.1	27.9	60.0	11.5
16	24.9	27.8	27.8	29.5	16.1	29.1	37.6	0.0	25.6	25.6	0.0	42.3
≥17	61.8	55.6	65.5	54.1	54.8	62.1	41.9	50.0	64.1	41.9	40.0	46.2
Household income (USD^e^/year), %												
<$50,000	4.8	5.6	4.0	6.6	19.4	7.8	2.6	25.0	2.6	9.5	20.0	0.0
$50,000–$99,999	21.7	29.4	24.4	29.5	29.0	24.5	21.1	25.0	20.5	26.2	40.0	11.5
$100,000–$149,999	35.7	29.4	33.7	21.3	19.4	31.3	36.0	0.0	20.5	30.1	40.0	23.1
≥$150,000	37.8	35.3	38.0	42.6	32.3	36.3	40.4	50.0	56.4	33.3	0.0	53.8
Race/ethnicity, %												
Non-Hispanic white	83.8	72.2	80.4	85.3	77.4	87.4	81.2	100.0	79.5	72.1	60.0	80.8
Non-Hispanic Black	2.4	5.6	5.0	3.3	6.5	0.97	5.1	0.0	5.1	0.0	0.0	0.0
Non-Hispanic Asian	4.7	5.6	3.9	1.6	0.0	3.9	2.6	0.0	5.1	0.0	20.0	3.9
Non-Hispanic other^f^	2.4	0.0	3.2	0.0	0.0	1.9	2.6	0.0	7.7	4.7	20.0	7.7
Hispanic	6.7	16.7	7.5	9.8	16.1	5.8	8.6	0.0	2.6	9.3	0.0	7.7
Urbanicity^g^, (%)												
Rural	0.7	0.0	0.0	3.3	3.2	2.9	0.0	0.0	0.0	4.7	0.0	3.9
Urban Cluster	0.7	0.0	0.0	13.1	9.7	8.7	0.9	0.0	0.0	16.3	0.0	11.5
Urban	98.7	100.0	100.0	83.6	87.1	88.4	99.2	100.0	100.0	79.1	100.0	84.6
Employed, (%)	92.9	83.3	93.2	91.8	87.1	89.3	94.0	75.0	94.9	95.4	100.0	96.2
Hours of Work/week (Median, IQR)	40 (35–40)	40 (20–40)	40 (36–41)	40 (36–40)	40 (35–40)	40 (32–40)	40 (40–50)	40 (20–42.5)	40 (40–45)	40 (40–45)	46 (42–50)	41 (40–48)
Current Smoker, (%)	2.5	0.0	1.1	1.6	0.0	1.0	1.7	0.0	0.0	4.7	0.0	0.0
Parous, (%)	43.7	61.1	42.3	47.5	41.9	45.6	–	–	–	–	–	–
Ever Impregnated a Partner (%)	–	–	–	–	–	–	40.0	25.0	28.2	52.4	0.0	28.0
History of Infertility (%)	3.8	5.6	3.6	3.3	0.0	10.7	–	–	–	–	–	–

^a^ Participant was eligible to participate in the protocol but did not consent to participate after invitation via email.

^b^ Participant consented to participate but did not complete the protocol by either not showing up to the in-clinic appointment, or coordinating the return of biospecimens.

^c^ Participant collected all biospecimens and was compensated for their efforts.

^d^ Interquartile Range.

^e^ United States Dollars.

^f^ Other includes those who identify as mixed-race, Native American or Pacific Islander, and Middle Eastern or North African.

^g^ “Urban” refers to residing within a United States census tract with 50,000 people or more, “Urban Clusters” refers to residing within a United States census tract of at least 2,500 and less than 5,000 people “Rural” encompasses all census tracts not included within an urban area.

Correct headers:

**Table 1:** Characteristics of enrolled E-PRESTO participants stratified by sex, protocol, consent, and successful completion of the protocol.

Characteristic	Female participants						Male participants					
	In-clinic protocol			Mail-based protocol			In-clinic protocol			Mail-based protocol		
	Did not consent^1^ (*n* = 450)	Consented but did not complete^2^ (*n* = 10)	Consented and completed^3^ (*n* = 279)	Did not consent (*n* = 61)	Consented but did not complete (*n* = 31)	Consented and completed (*n* = 103)	Did not consent (*n* = 117)	Consented but did not complete (*n* = 4)	Consented and completed (*n* = 37)	Did not consent (*n* = 43)	Consented, but did not complete (*n* = 5)	Consented and completed (*n* = 26)

The authors apologize for this error and state that this does not change the scientific conclusions of the article in any way. The original article has been updated.

